# Indian adolescents’ perceptions of anaemia and its preventive measures: Aqualitative study

**DOI:** 10.1017/jns.2024.4

**Published:** 2024-02-16

**Authors:** Neha Rathi, Sangeeta Kansal, Aryan Raj, Nikitha Pedapanga, Immanuel Joshua, Anthony Worsley

**Affiliations:** 1 Department of Home Science, Mahila Mahavidyalaya, Banaras Hindu University, Varanasi 221005, Uttar Pradesh, India; 2 Department of Community Medicine, Institute of Medical Sciences, Banaras Hindu University, Varanasi 221005, Uttar Pradesh, India; 3 Department of Psychiatry, Government Medical College and Hospital, Chandigarh 160030, India; 4 School of Exercise and Nutrition Sciences, Deakin University, Geelong, VIC 3220, Australia

**Keywords:** India, Adolescents, Interviews, Anaemia

## Abstract

High prevalence of anaemia is a severe public health problem in several low- and middle-income countries like India. A qualitative inquiry was designed to understand the perceptions of adolescents regarding anaemia and anaemia prevention measures. Convenience sampling was employed to recruit 39 adolescents (19 girls; 20 boys) from Tikari, India. Interviews were carried out in the local language, audio-recorded and transcribed verbatim. Hemoglobin concentration was also assessed from a single drop of capillary blood using the HemoCue, and the participants were asked to share their Science/Biology and Home Science textbooks. Interview data was analysed thematically. Descriptive statistics were used to examine the distributions of the hemoglobin data while textbooks were analysed using content analysis to verify the coverage of anaemia and anaemia-related matter. Seven themes were identified: (i) Poor understanding of the term anaemia; (ii) Minimal discussion about anaemia in classroom; (iii) Limited knowledge about symptoms of anaemia; (iv) Limited awareness about prevention and cure of anaemia; (v) Perception of iron folic acid and deworming tablets among students; (vi) Lack of contribution of health workers in the prevention of anaemia; (vii) No knowledge of ‘Anemia free India’ programme. More than half of the sample had anaemia (16.7% mild anaemia, 33.3% moderate anaemia, 2.8% severe anaemia). Content analysis revealed that there was limited discussion about anaemia in both Home Science and Science textbooks. Behavioural interventions should focus on inculcating healthy culinary and dietary practices and addressing the gaps in knowledge and understanding of anaemia and its prevention among adolescents.

## Introduction

For several decades, anaemia has continued to be a major public health concern in India with almost 50% of its children and adolescents being afflicted^(1,2)^. Owning to its multifactorial aetiology, the prevalence of anaemia among Indian adolescents is alarmingly high^(2,3)^. The Comprehensive National Nutrition Survey (CNNS, 2016-18) reported that 18% and 40% of the adolescent (10-19 years) boys and girls had anaemia^(3)^. A higher prevalence of anaemia was observed in older adolescents aged 15-19 years (F:48%; M:18%) when compared to their younger counterparts aged 10-14 years (F:32%; M:17%)^(3)^.

Anaemia is considered to be caused by a range of factors including deficiencies of micronutrients like iron, folic acid, vitamin B_12_, vitamin A, copper, zinc, worm infestations, genetic blood disorders, inflammation, and other physiological conditions like pregnancy^(2–4)^. Anaemia arising from micronutrient deficiencies can be addressed through school-based food literacy interventions which have the potential to raise awareness about appropriate dietary practices among adolescents^(5)^. Anaemia may reduce infection resistance among adolescents, impair their physical growth and mental development, diminish their physical fitness, work capacity, and scholastic performance^(6–8)^. Importantly, when adolescent girls with anaemia conceive, they are subjected not only to the risk of maternal morbidity and mortality but also the threat of premature birth, low birth weight, and perinatal mortality^(6,9)^. Moreover, babies born to mothers with anaemia have an increased risk of anaemia in the first six months of life^(10)^.

In light of these adverse health consequences and the enduring prevalence of anaemia, several prevention programmes have been launched by the Government of India. One of the most current programmes is the Anemia Mukt Bharat (AMB, i.e. Anemia Free India) programme launched in 2018 with the aim of reducing the nationwide prevalence of maternal and child anaemia. Informed by the learning and experiences from previous health programmes including the NIPI (National Iron Plus Initiative) and consistent with international best practices, AMB is fundamentally a 6 × 6 × 6 strategy on iron supplementation^(11,12)^. First, it focusses on six beneficiary groups comprising (i) children aged 6-59 months; (ii) children aged 5-9 years; (iii) adolescent boys and girls aged 10–19 years; (iv) women of reproductive age; (v) pregnant women; and (vi) breastfeeding mothers^(11)^. Second, six institutional mechanisms were developed to reinforce accountability and implementation of the programme: (i) intra-ministerial coordination; (ii) national AMB unit; (iii) merger with other ministries; (iv) bolstering of public health supply chain and logistics; (v) advanced research on anaemia control; and (vi) creation of AMB dashboard and digital portal^(11)^. Lastly, the six interventions planned under AMB are: (i) prophylactic iron folic acid (IFA) supplementation; (ii) deworming; (iii) intensified year-round behaviour change campaigns including confirming delayed cord clamping in neonates; (iv) use of digital methods (e.g. digital hemoglobinometers) for anaemia testing and point of care treatment; (v) mandatory delivery of IFA-fortified foods in various public health programmes; and (vi) remedy for non-nutritional causes of anaemia in endemic pockets with specific focus on malaria, fluorosis, and hemoglobinopathies^(11)^. Besides AMB, the Weekly Iron Folic Acid Supplementation (WIFS) is also a popular government-based health programme for anaemia eradication^(13)^. WIFS currently serves 112 million adolescents aged 10–19 years including 84 million in-school and 28 million out-of-school beneficiaries^(13)^. It involves administration of supervised weekly supplement of 100 mg elemental iron and 500 µg folic acid adopting a fixed day approach. Biannual deworming for control of intestinal worm infestation and counselling for improving food intake are also delivered as part of WIFS^(13)^.

However, despite various government programmes like the AMB and WIFS supplying IFA tablets, anaemia continues to be a serious public health problem in India^(3,8,14)^. Moreover, since the launch of the AMB strategy in 2018, the prevalence of anaemia has increased from 54% in 2015–2016 to 59% in 2019–2021 in adolescent girls and similarly in adolescent boys from 29% in 2015–16 to 31% in 2019–2021.^(15)^.

To accelerate reductions in anaemia prevalence, a strong push to nurture anaemia reduction behaviours at the individual, family, and household levels is essential along with effective IFA supplementation^(16)^. For designing behaviour change interventions, a comprehensive understanding of beneficiaries’ perceptions of anaemia, its causes, consequences, and preventive measures is necessary^(16)^. Regrettably, there is a dearth of literature on Indian adolescents’ perceptions about anaemia and the ongoing AMB programme. Moreover, most of the available evidence is limited to pregnant women and adult populations^(16)^. The existing literature on anaemia is devoid of in-depth qualitative data. Furthermore, the views of adolescent boys on anaemia are relatively underrepresented in the current literature. Since most adolescent boys will become fathers in future, it is imperative for them to know the implications of anaemia for the newborn. This dearth of information warrants the need to investigate the perceptions of Indian adolescent boys and girls about anaemia and AMB. Exploring adolescents’ perceptions is a crucial step towards understanding the contextual factors that influence adolescents’ health-seeking behaviours about anaemia. Increased knowledge regarding underlying motivations for behaviour change would help public officials and public health practitioners to design feasible, sustainable, cost-effective, and targeted social norm-based interventions^(17,18)^ to diminish anaemia prevalence in adolescents. Therefore, this qualitative inquiry was designed to provide first-hand information on the views of vulnerable Indian adolescents about anaemia and the AMB which can potentially assist in strengthening and modifying the continuing AMB programme in order to achieve its maximum potential.

## Methods

### Study design

A social constructivist perspective^(19)^ was adopted for the present study with a focus on the interviewees’ lived experiences around the topic of interest. A qualitative research methodology^(20,21)^ was implemented using interviews to explore adolescents’ perceptions about anaemia and anaemia prevention strategies. This qualitative inquiry was conducted according to the guidelines laid down in the Declaration of Helsinki, and all procedures involving human subjects were approved by the Institutional Ethical Committee at Institute of Medical Sciences, Banaras Hindu University (Dean/2022/EC/3410). Written informed consent was obtained from all subjects and their parents. The Consolidated Criteria for Reporting Qualitative Research (COREQ) – a 32-item checklist^(22)^ was used to comprehensively report all the aspects of this qualitative inquiry.

### Research team

All the interviews were carried out by a female post doctoral researcher (NR) with a background in Home Economics and a PhD in behavioural nutrition and with substantial experience in qualitative research. NR has previously conducted a number of qualitative research inquiries^(23–26)^ where she interviewed both male and female participants. Moreover, there is no evidence that having male interviewer would make any difference to the boys’/men’s responses. During the interviews, NR was supported by a male note keeper (AR) with a Master’s degree in social work. He transcribed all the interviews as well as assisted in coding. A female medical doctor (NP) collaborated in the data collection process. She used the HemoCue portable hemoglobin photometer (HemoCue Hb 301 Analyzer, Angelholm, Sweden) to determine the hemoglobin levels of the interviewees. She further contributed to the research process by translating all the transcripts in Hindi to English. A male physician specialising (IJ) in family medicine assisted with the content analysis of the Science/Biology and Home Science textbooks. The remaining two researchers, that is, a female medical doctor (SK) with expertise in community medicine and a retired male food psychologist (AW) were involved in the conception of the study and its supervision. The contribution of both male and female authors from varied disciplines reduced the possibility of any gender or professional biases affecting the data analysis.

### Study setting and sample

Convenience sampling was used to recruit study participants from Tikari village (Tikari is located in Varanasi district in the state of Uttar Pradesh; Uttar Pradesh is the most populous state of India). Adolescents aged 10-19 years formed the study sample because the national level survey data from the state of Uttar Pradesh suggests that 29.9% of adolescent boys (< 13.0 g/dl) and 52.9 % of girls (< 11.0g/dl) aged 15–19 years in rural areas were diagnosed with anaemia^(15)^. In addition, adolescents aged 10–19 years are the direct beneficiaries of the AMB campaign further endorsing our selection of adolescents aged 10–19 years^(11)^. A social worker from the Department of Community Medicine of the Institute of Medical Sciences, Banaras Hindu University, where the present research was designed and conducted, helped to identify the houses in which adolescents lived. Subsequently, the lead author (NR) met the adolescents and their parents in person to explain to them about the research inquiry as well as seek their consent for participation in the interviews.

### Data collection

Based on the research aim and previous literature on anaemia, an interview topic guide comprising open-ended questions was developed. It was pre-tested with six adolescents for comprehensibility and content. Minor amendments to the interview guide were made, for example, a change in the sequence of questions. The data from the pre-test was not merged with the main data collection.

Using the interview guide (Table 1), semi-structured, one-to-one interviews were carried out by the lead author (NR) between November 2022 and January 2023. At the onset of the face-to-face conversations, the interviewer (NR) provided a brief overview of the project and assured the interviewees of their right to confidentiality^(27)^ as well as seeking their approval for audio recording of the interactions^(27)^. Interviewees were further notified that their participation was completely voluntary and that they were free to withdraw from the research project at any point before data preparation as once the data is synthesised it will not be possible to identify responses. None of the participants shared any kind of personal or professional associations with the researchers. Besides the questions listed in the interview guide, a few probing questions were also introduced. For example, if the adolescent responded ‘No’ to ‘Have you heard about the term “anemia”?’ then he/she was asked whether he/she had ever heard about the concept ‘lack of blood in the body’.

**Table 1.** Interview guide

Q1	Have you heard about the term ‘anemia’?
Q2	What do you know about anaemia?
Q3	Do you think it is important to know about anaemia at this age?
Q4	Are you aware of the Anemia Mukt Bharat Program? (Probe questions – Do you receive IFA tablets? Does your school provide deworming tablets? Does ASHA visit your home and give IFA tablets?)

Recruitment of adolescents lasted until data saturation^(28)^ was reached as the researcher began to hear similar views from new interviewees, and no new concepts were emerging in the data. All the interviews were conducted in the local language and digitally recorded. The interviews were carried out in a quiet and open area in the village where no individual known to the interviewee was present. Interview duration varied from 13 minutes to 24 minutes. Upon completion of the interviews, the interviewees were requested to share their Science/Biology and Home Science (i.e. Home Economics/Family and Consumer Sciences) textbooks for the purpose of content analysis. Content analysis was undertaken to identify any fallacies within the school curriculum regarding anaemia. Home Science and Science/Biology textbooks were chosen as nutrition-related topics are covered in these two disciplines only^(26,29,30)^. The note keeper (AR) took photographs of the relevant chapters where anaemia and anaemia-related information was mentioned and/or discussed.

In addition, adolescents were asked for permission to estimate their hemoglobin levels through the consent form. Nineteen boys and 17 girls provided consent and NP assessed their hemoglobin concentration through the prick method. The hemoglobin was assessed from a single drop of capillary blood using the HemoCue Hb 301 photometer^(31)^ which has minimal risk to the respondent. NP had received prior training in using the HemoCue. The instrument was factory calibrated against the international reference method for hemoglobin determination, and therefore no further standardisation was required. Blood collection was carried out in accordance with the norms specified in the user manual, that is, at room temperature and ground level. Basic socio-demographic information was sought such as age, gender, year level at school and whether adolescents study Science/Biology and/or Home Science in school. All the respondents received plain yoghurt and seasonal fruits like banana, guava, and orange for their participation in the present inquiry.

### Data analysis

Following data collection, all interview data (i.e. audio recordings) and photographs of the book chapters were kept in a password protected drive folder. All recordings were transcribed verbatim and translated to English. Post transcription, member checking was done for assessing fidelity wherein all participants were invited to review the transcripts, but none showed interest in doing so. The transcribed data were analysed using the NVivo 12 software program (QSR International Pvt Ltd. 2010) to identify different, interrelating themes through an approach underpinned by template analysis technique^(27)^. In this technique, both inductive (codes are generated directly from the raw data) and deductive (codes are derived from the review of literature and research questions) coding are implemented to form a template (i.e. a set of codes) wherein themes are developed in line with the research objective and in relation to investigators’ interpretations of the data. Two analysts (NR, AR) independently developed the code set and in case of difference of opinion, the code set was reviewed and modified through mutual discussion so that the final code set was illustrative of the transcribed data^(19)^. Additionally, to confirm inter-rater reliability, a psychologist and one dietician were invited to independently review and analyse six transcripts which were previously coded by the primary analysists (NR, AR)^(32)^.

Descriptive statistics were used to examine the distributions of the hemoglobin data. The WHO classification of anaemia was used to define anaemia severity among adolescents^(33)^ (Table 2). A content analysis technique^(34,35)^ was used for analysing the book chapters to verify the coverage of anaemia and anaemia-related content. Content analysis is a widely used qualitative research technique for analysing the content of a variety of data, such as visual and verbal data^(34)^.

**Table 2.** Anaemia severity classification (Hemoglobin values in g/dL)^a^

	Non anaemia	Mild anaemia	Moderate anaemia	Severe anaemia
Girls	≥ 12	11-11.9	8-10.9	< 8
Boys	≥ 13	11-12.9	8-10.9	< 8

a ^(33)^.

## Results

Twenty boys and 19 girls aged 10-19 years took part in the interview. Out of these 39 adolescents, 38 were studying between 6th and 12th grades while one female adolescent was an early school leaver. Thirty-seven adolescents were studying in government schools while one was attending a private school. Twenty-eight adolescents were studying Biology/Science in school while only 12 female adolescents were studying Home Science. The mean age of the adolescents was 14.35 years (SD= 2.70).Thirty-six adolescents (19 boys; 17 girls) provided consent for hemoglobin estimation. The mean hemoglobin levels in boys and girls were 12.8 g/dl and 11.1 g/dl respectively^(33)^. More than half of the sample had anaemia (16.7% mild anaemia, 33.3% moderate anaemia, 2.8% severe anaemia; Table 3).

**Table 3.** Anaemia classification among the study sample based on hemoglobin estimation^a^

		Anaemia classification^a^				Total
		Non anaemia	Mild anaemia	Moderate anaemia	Severe anaemia	
Gender	F	5	2	9	1	17
		29.4%	11.8%	52.9%	5.9%	100.0%
	M	12	4	3	0	19
		63.2%	21.1%	15.8%	0.0%	100.0%
Total		17	6	12	1	36
		47.2%	16.7%	33.3%	2.8%	100.0%

a ^(33)^.

Seven themes were identified through analyses of 39 interviews associated with anaemia and anaemia prevention strategies: (i) Poor understanding of the term ‘anemia’; (ii) Minimal discussion about anaemia in classroom; (iii) Limited knowledge about symptoms of anaemia; (iv) Limited awareness about prevention and cure of anaemia; (v) Perception of IFA and deworming tablets among students; (vi) Lack of contribution of health workers towards prevention of anaemia; (vii) No knowledge of Anemia Mukt Bharat (AMB). These themes along with illustrative quotes are discussed below:

### Theme 1: Poor understanding of the term ‘anemia’

The majority of the sample claimed that they were not aware of the term ‘anemia’. However, they noted that they were aware of the phrase ‘khoon ki kami’ which means lack of blood. In addition, there were few interviewees who had neither heard about anaemia nor ‘khoon ki kami’.

*‘No Mam I have never heard about anemia…I did hear about khoon ki kami (i.e. lack of blood in the body) once. It causes weakness. Papa said it once…’* (A25, 12 years, F)

*‘No Mam I don’t know what anemia is….Also never heard about khoon ki kami.’* (A28, 13 years, M)

Nevertheless, some students claimed that they had only heard the term ‘anemia’ but had no knowledge about what it is. Only two adolescents claimed that anaemia is a nutritional deficiency which is characterised by low hemoglobin level.

*‘Yes Mam…I only heard the term anemia, but I don’t know in detail… Mam it was written in the book I read in school….I don’t remember the name of the book.’* (A29, 15 years, M)

*‘Yes, I have heard of anemia. It is khoon ki kami. I have studied about it in Science class….In anemia we get tired, headache is there, and we have low hemoglobin level.’* (A38, 13 years, F)

*‘Khoon ki kami is iron deficiency which we can cure by having one iron tablet per week’* (A23, 18 years, M)

### Theme 2: Minimal discussion about anaemia in classroom

Mixed responses were recorded regarding discussion of anaemia in classrooms. Some adolescents reported that they were taught about anaemia either in Science or Home Science classes, but it was not discussed in detail. Two female adolescents mentioned that they recently came to know about anaemia through a special programme on anaemia which was organised in their school by a non-government organisation (NGO) on Children’s Day (In India Children’s Day is celebrated on 14^th^ November every year).

*‘Anemia is a disease in which our body becomes weak, and I have heard in 8* ^*th*^ *class…Mam told us in Science class…’* (A34, 16 years, M)

*‘Yes Mam, I heard about anemia in school on Children’s day on 14th. Some visitors had come, and they told us that if people want to stay healthy, they should eat green vegetables, black gram, jaggery….’* (A14, 17 years, F)

However, some students complained that the school curriculum did not include topics about anemia, and therefore they were ignorant about it.

*‘In school it (anemia) is never discussed. Mam, I heard about anemia in hospital.’* (A9, 13 years, M)

### Theme 3: Limited knowledge about symptoms of anaemia

Despite limited knowledge about anaemia, some adolescents were aware of a few common symptoms of anaemia which included weakness and dizziness. Other symptoms like headache and pale eyes and nails were cited less frequently by the interviewees. Mostly family members informed the adolescents about the symptoms as noted during the interviews.

*‘Due to ‘khoon ki kami’ humans become very weak and lean…. Mom and Dad told me about it.’* (A7, 12 years, M)

*‘In anemia our eyes become white, our nails become white, and we get swelling in our legs, like this many symptoms happen but it mainly occurs in pregnant women…’* (A14, 17 years, F)

### Theme 4: Limited awareness about prevention and cure of anaemia

Some adolescents reported that consumption of green leafy vegetables, black gram, jaggery, and IFA tablets can cure anaemia. Only two female adolescents noted that food should be cooked in iron vessels to enhance the iron content. This theme is reflected in the quotes below:

*‘Yes, we can cure it (anemia) if we eat leafy vegetables and vegetables which have high level of iron content it, then it (anemia) will get cured.’* (A38, 13 years, F)

*‘…Green curry should be made in an iron pan as it will give iron.’* (A13, 17 years, F)

*‘We can cure iron deficiency by taking one iron tablet per week.’* (A23, 18 years, M)

### Theme 5: Perception of IFA and deworming tablets among students

A large proportion of the sample (n=23) claimed that IFA tablets were provided by the school authorities however there was no stipulated time for its distribution, that is, according to some adolescents it is distributed once in a month or two months while others noted weekly or fortnightly distribution of IFA.

*‘We get iron tablets every three months, and the slip has 10 pills.’* (A32, 19 years, M)

*‘Yes, Mam iron tablet is available in school. Once a month it is given to us. I eat it sometimes. I keep it at home because it tastes weird.’* (A13, 17 years, F)

*‘I got the iron tablet in school. Previously I used to get it, now I won’t. In 8* ^*th*^ *grade I used to get it.’* (A4, 12 years, M)

Despite receiving the IFA tablets, quite a few of the adolescents refrained from consuming them for various reasons which included not being aware of the significance of consuming IFA tablets, being scared of any health implications arising from consuming them, forgetfulness, aversion to medicines, and self-perception of being disease free.

*‘Mam (school teacher) will just distribute it (iron tablet), why will she tell anything. I will not eat it…Because I don’t know for what reason should I eat it…So, I throw it away. We usually get this in one or two years.’* (A7, 12 years, M)

*‘No Mam, they (school authorities) only give iron medicine, but I never had it till now. Our teacher gave us the medicine, a girl had it and she vomited so since then I didn’t have it as I was scared.’* (A15, 17 years, F)

*‘Weekly we get iron tablet, but I don’t eat it…There is no particular reason. I just don’t feel like eating it!’* (A39, 15 years, F)

Only a few participants reported that they received deworming tablets in school, and mixed views were reported with regards to the time of delivery.

*‘For stomach worms and iron, we get medicines in school. I exactly don’t remember when I received it last but whenever they (school authorities) give, I eat it.’* (A18, 11 years, M)

*‘Worm medicines are available in school. Many days have passed since we got them’* (A10, 10 years, F)

### Theme 6: Poor engagement of health workers in the prevention of anaemia

Thirty-two study respondents criticised the lack of engagement of community health workers, that is, ASHAs (Accredited Social Health Activist) and AWWs (Anganwadi Worker) in preventing anaemia at the grassroots level. It was reported that health workers rarely visited the adolescents’ homes and subsequently did not provide IFA and deworming tablets. The adolescents further noted that health workers did not organise any meetings to discuss anaemia. This criticism is highlighted in the following three quotes:

*‘ASHA didi (i.e. sister) don’t give any tablets now but they gave before COVID-19. They never talked about anemia in the village.’* (A32, 19 years, M)

*‘AWW used to give medicines to increase blood rate around one year ago….Now they don’t give.’* (A25, 12 years, F)

*‘Yes, they (ASHA) give medicines for stomach worms. They give it to children younger than me….They did not call us for any meeting on anemia….’* (A28, 13 years, M)

### Theme 7: Little knowledge of AMB

With the exception of three respondents, none of them were aware of the AMB programme launched by the Government of India in 2018. Those who were aware mentioned that they were informed about the programme at a special function organised by an NGO on the occasion of Children’s Day.

*‘I don’t listen to the news, so I don’t know about this program (AMB)’* (A30, 15 years, M)

*‘Yes, Mam I have heard about AMB a few days back. There was a program on Children’s Day in the school on 14 November and there it was mentioned….’* (A13, 17 years, F)

*‘I have no clue about this program (AMB). Never heard it in school!’* (A17, 11 years, M)

Content analyses of Home Science and Science/Biology textbooks used in schools recognised by the U.P. Board of High School and Intermediate Education suggest very limited discussion about anaemia (Table 4). For example, there is no discussion about anaemia in Science textbook used in seventh grade. In the same vein, barely any information on anaemia is available in Science/Biology textbooks implemented between 9^th^ and 12^th^ grades. For instance, only the name ‘Sickle Cell Anemia’ was mentioned in the Genetics chapter of Grade XII Biology textbook. Home Science was only offered to female students while Science and Biology were offered to both boys and girls.

**Table 4.** Findings of content analyses of Home Science and Science/Biology^a^ textbooks

Grade/Subject	The term ‘anemia’	Khoon ki kami (Hindi translation of anaemia)	Food sources of iron	Symptoms of anaemia	Causes of anaemia
6^th^/Science	Yes	Yes	Yes	Yes	Yes
6^th^/Home Science	Yes	No	Yes	Yes	Yes
7^th^/Science	No	No	No	No	No
7^th^/Home Science	Yes	No	Yes	Yes	Yes
8^th^/Science	Yes	No	Yes	No	No
8^th^/Home Science	Yes	No	No	No	Yes
9^th^/Science	No	No	No	No	No
9^th^/Home Science	Yes	Yes	No	Yes	Yes
10^th^/Science	No	No	No	No	No
10^th^/Home Science	No	No	No	No	No
11^th^/Biology	No	No	No	No	No
11^th^/Home Science	Yes	No	No	Yes	Yes
12^th^/Biology	Yes	No	No	No	No
12^th^/Home Science	No	Yes	No	Yes	No

a Till Grade 10 all three Science subjects, that is, Physics, Chemistry and Biology are discussed in one textbook which is the Science textbook. In Grades 11 & 12, Physics, Chemistry, and Biology are taught as individual subjects and therefore have individual textbooks.

## Discussion

The present inquiry explored the views of both male and female adolescents on anaemia and its prevention in India. Besides exploring the adolescents’ perceptions, this study also assessed the hemoglobin levels of the participants as well as the anaemia content discussed in school textbooks. There was low awareness about anaemia and the ‘Anemia free India’ programme (AMB) among the participants. The adolescents noted that there was an irregular supply of IFA and deworming tablets in schools. They further criticised the lack of contribution of health workers in anaemia reduction. Anaemia was neither an integral part of the school curriculum nor textbooks and the hemoglobin field tests results suggested that more than half of the sample had anaemia.

The majority of the respondents were not aware of the term ‘anemia’. They had limited knowledge of the disease or its symptoms. This poor awareness was also observed in female adolescents in both Indonesia^(36)^ and southern India^(37)^. With regards to anaemia prevention, only two female interviewees talked about cooking food in iron vessels, and five girls mentioned consumption of iron-rich dietary sources and IFA tablets in preventing and treating anaemia. Some of these preventive measures were reported by school girls in a cross-sectional study conducted in New Delhi, India^(38)^. Disappointingly, these practices were not implemented by the girls in the Delhi-based study^(38)^. This underscores the need to enhance both declarative as well as procedural nutrition knowledge of adolescents through implementation of skill-based food and nutrition programmes in schools^(30)^. Indeed, food and nutrition education has been useful in correcting the dietary habits of children and adolescents^(39)^ which can considerably improve iron intake and consequently reduce iron deficiency anaemia^(40,41)^.

Adolescents’ poor awareness of anaemia could be attributed to limited discussion about anaemia in classrooms as reported by our adolescents. Generally, Indian schools do not prioritise nutrition education as noted in both qualitative^(26)^ and quantitative studies^(42,43)^. Furthermore, the content analysis of school textbooks revealed that anaemia-related topics were included only in some textbooks. In the same vein, Subba Rao and colleagues found that nutrition course content of Science books used in Indian schools (Grade I – X) is low^(44)^. They further noted that nutrition-related topics got around 10% coverage in textbooks offered to students in Grades I to VII, while they were omitted in subsequent grades^(44)^, a finding which resurfaced in the present study where anaemia was barely discussed in Biology/Science books from Grade IX onwards.

Additionally, Home Science was only offered to female students, a finding consistently reported in the past^(26,30,42)^. This selective learning could possibly lead to differences between male and female pupils in their anaemia-related knowledge. Possibly, this could be the reason that our female participants were better informed about anaemia-preventive dietary practices, that is, cooking in iron pots. Despite being better informed, the female participants reported higher prevalence of anaemia when compared to their male counterparts suggesting that the anaemia-preventive dietary practices were not performed by our female respondents. Nonetheless, despite these apparent dietary differences, both the male and female interviewees shared similar views on anaemia and AMB.

Another matter of concern was the poor supply of IFA and deworming tablets in schools as reported by the study participants. In line with these findings, Singh and coworkers found that only 2.8% of school (government) going girls (n=210) consumed IFA in the previous year to their survey and only 3.8% had consumed deworming tablet in last six months^(38)^. This could be attributed to ineffective implementation of the AMB programme. Indeed, the public health supply chain for IFA supplementation under the AMB programme has received harsh criticism in the recent past^(12,45)^. For instance, Ahmed and colleagues highlighted a number of shortcomings in the delivery of AMB services which included lack of fixed distribution schedule; absence of inventory management techniques and integrated management information system, and inadequate availability of transport vehicles^(45)^. Parallel to these findings, Joe and coworkers also found that the IFA supplementation coverage was relatively low among target groups including adolescents who were being supplied through a multi-departmental convergence mechanism (e.g. health and education department)^(12)^. The inefficiencies in the IFA supplementation coverage could be eliminated through active leadership and involvement of all stakeholders across government departments, for example, Health, School Education as well as development and implementation of clear and well-defined interventions focusing on specific bottlenecks in the IFA supply chain^(12,45)^.

Another criticism raised about AMB was that community health workers neither provided any iron tablets nor did they discuss anaemia or AMB with the adolescents. In contrast, a recent review suggests that ASHAs and AWWs are responsible for improving adolescents’ health status through distribution of IFA and deworming tablets, promoting menstrual hygiene schemes, conducting preventive health check-up camps and dietary counselling^(46)^. However, both ASHAs^(47)^ and AWWs^(48)^ report that they often feel overworked and underpaid. Finally, the findings from the hemoglobin field tests revealed that nearly three-fourths of the adolescent girls had anaemia while two-fifths of the boys were diagnosed with anaemia. In this light, both school and home-based nutrition interventions should be developed which focus on leveraging adolescents’ food agency^(49,50)^ to improve their hemoglobin levels. Derived from Social Cognitive Theory (SCT), agency refers to the ability to intentionally produce effects by one’s actions^(51)^. Indian adolescents can enhance their agency in cooking, grocery shopping and food consumption through implementation of skills-based interventions.

Theory-driven interventions are associated with larger intervention effects^(52)^. Theoretical models like the Health Belief Model, Transtheoretical Model (TTM), SCT, and Theory of Planned Behavior have been largely used to inform behaviour change interventions^(53,54)^. A recent systematic review highlights TTM as a successful theoretical framework for nutrition interventions aiming at improving dietary intake in adolescents^(54)^. Most of the successful TTM-driven nutrition interventions involved digital technology which included the use of websites, videos, SMS (Short Messages Service) messages as means of disseminating information regarding fruit and vegetable intake, low-fat diet, and culinary skills^(54)^. Perhaps, TTM can form the theoretical basis of future sex- and age-based tailored anaemia reduction interventions, and these interventions can be delivered through various information and communication technologies like social media, television, and radio to facilitate preparation and consumption of micronutrient-rich recipes among adolescents.

### Strengths and limitations

To the best of authors’ knowledge, this is the first time that an in-depth research inquiry has been undertaken to explore the perceptions of both male and female adolescents about anaemia and its prevention in India. In addition, there is paucity of data available on adolescents’ views about anaemia particularly in rural areas either in India or other developing economies. Thus, the emerging evidence has important public health implications both for India and other low- and middle- income countries. Moreover, the use of a content analysis technique in evaluating the Science/Biology and Home Science textbooks was a novel approach in anaemia research. In addition, the inclusion of both male and female adolescents further strengthened our study findings as previous investigations have largely focused on females, particularly pregnant women, while ignoring the critical views of male respondents.

Despite these strengths, this study has some limitations. First, the use of convenience sampling could have compromised the generalisability of our findings. However, the use of deliberate sampling techniques like convenience sampling is emphasised in the qualitative research literature since the aim of qualitative research is not to generalise but to generate first-hand, comprehensive knowledge about the subject matter^(55,56)^. Second, the interviewees could have provided socially desirable responses, nonetheless, the interviewees openly discussed the irregular supply of IFA and deworming tablets and the limited discussion of anaemia in classroom suggesting that socially desirable responses were limited in this study. Another significant limitation could be associated with hemoglobin estimation using single drop of capillary blood as recent evidence prohibits the use of this method to diagnose anaemia because drops of capillary blood produces too much random variation to approximate true hemoglobin values^(57)^.

### Conclusion

Overall, our findings reflect the views of a sample of rural Indian adolescents regarding anaemia and government-led anaemia prevention strategies. These views strongly endorse the need for revision of both Home Science and Science/Biology curricula and textbooks used in Indian schools to empower adolescents with declarative as well as nutrition knowledge. In addition, policymakers and public health professionals should design more effective school-based behavioural nutrition interventions, for example, building kitchen gardens in schools, culinary workshops, integrating food studies with other subjects like Mathematics, Geography etc. to curb the escalation of anaemia among adolescents. The aim of these school-based behavioural programmes should be to cultivate culinary skills and healthy eating habits and also address adolescents’ gaps in knowledge of anaemia. Underpinned by theoretical models, these interventions should also focus on leveraging adolescents’ agency to increase their hemoglobin levels through preparation and consumption of iron-rich recipes. With regards to the government-led anaemia eradication programme, it is vital to harness synergy in IFA supplementation coverage and the overall AMB programme implementation across different departments, for example, Health, School Education through robust training and sensitisation workshops of all the stakeholders. With all these efforts, it should be possible to make India anaemia free!

## Supporting information

Rathi et al. supplementary material Rathi et al. supplementary material

## Supplementary material

The supplementary material for this article can be found at https://doi.org/10.1017/jns.2024.4
