# Indian adolescents’ perceptions of anaemia and its preventive measures: A qualitative study – ERRATUM

**DOI:** 10.1017/jns.2024.12

**Published:** 2024-02-28

**Authors:** Neha Rathi, Sangeeta Kansal, Aryan Raj, Nikitha Pedapanga, Immanuel Joshua, Anthony Worsley

The publisher regrets that this article was published under the incorrect title of ‘Indian adolescents’ perceptions of anaemia and its preventive measures: Aqualitative study’. This should have been ‘Indian adolescents’ perceptions of anaemia and its preventive measures: A qualitative study’.

The article has been updated to reflect this.

## References

[ref1] Rathi N , Kansal S , Raj A , Pedapanga N , Joshua I , Worsley A. Indian adolescents’ perceptions of anaemia and its preventive measures: A qualitative study. Journal of Nutritional Science. 2024;13:e9. doi: 10.1017/jns.2024.4 38379591 PMC10877140

